# Aloe emodin attenuates Aβ_1–42_-Induced neurotoxicity in SH-SY5Y cells via downregulation of PPM1K

**DOI:** 10.1515/med-2026-1473

**Published:** 2026-07-07

**Authors:** Fang Huang, Mingxing Guo, Yiran Yang, Zhizhong Wang

**Affiliations:** Department of Traditional Chinese Medicine, Wuhan Central Hospital, Tongji Medical College, Huazhong University of Science and Technology, Wuhan, 430014, China; College of Chinese Medicine, Hubei University of Chinese Medicine, Wuhan, 430033, China; Department of Neurosurgery, The Third People’s Hospital of Hubei Province, Affiliated Jianghan University, Wuhan, 430033, China

**Keywords:** Alzheimer’s disease, aloe emodin, PPM1K, Aβ1–42, SH-SY5Y

## Abstract

**Objectives:**

To study the role and underlying mechanism of aloe emodin, a natural anthraquinone compound known for antioxidant, and anti-inflammatory activities, in Alzheimer’s disease (AD).

**Methods:**

Aβ_1–42_-induced injury model was established using SH-SY5Y cells. A combination of CCK-8 assay, flow cytometry, ELISA, ROS fluorescence probe, RT-qPCR, and western blot assay was employed to systematically evaluate the effects of aloe emodin on cell viability, apoptosis, inflammation, and oxidative stress. Potential molecular targets were identified through bioinformatics analysis. The regulatory role of PPM1K in aloe emodin-mediated neuroprotection was further validated using PPM1K overexpression experiments.

**Results:**

Bioinformatics analysis revealed that PPM1K is significantly upregulated in AD and showed predicted in silico binding with aloe emodin. Aloe emodin markedly improved the viability of Aβ_1–42_-treated SH-SY5Y cells, suppressed apoptosis, reduced the release of pro-inflammatory cytokines, and attenuated ROS generation, accompanied by the downregulation of PPM1K expression. In contrast, overexpression of PPM1K significantly weakened the neuroprotective effects of aloe emodin, as evidenced by decreased cell viability and increased levels of apoptosis and oxidative stress.

**Conclusions:**

Aloe emodin alleviates Aβ_1–42_-induced neuronal injury by targeting and downregulating PPM1K expression, suggesting that PPM1K is a critical mediator of the neuroprotective effects of aloe emodin.

## Introduction

Alzheimer’s disease (AD) is the most common neurodegenerative disorder, clinically characterized by progressive memory loss, cognitive decline, and behavioral abnormalities, severely affecting the quality of life in the elderly [1]. With the acceleration of global population aging, the incidence of AD continues to rise, making it a major public health challenge worldwide [2]. Currently, clinical treatment of AD remains largely limited to symptomatic management, with no effective interventions available to halt or reverse disease progression [3]. Therefore, the development of novel therapeutic agents with multi-target and multi-pathway regulatory potential has become a key focus in AD research.

The hallmarks of AD include extracellular deposition of β-amyloid (Aβ) plaques and intracellular accumulation of hyperphosphorylated Tau protein forming neurofibrillary tangles (NFTs) [4],^5^]. Among various Aβ isoforms, Aβ_1–42_ is the most neurotoxic and has been shown to induce a cascade of deleterious events, including excessive production of reactive oxygen species (ROS), disruption of calcium homeostasis, mitochondrial dysfunction, inflammasome activation, and pyroptosis [6], [7], [8]. These processes collectively exacerbate neuronal apoptosis and synaptic impairment, making Aβ_1–42_ a central driver of AD progression.

Notably, mitochondrial dysfunction has been recognized as an early and critical event in the pathogenesis of AD, potentially occurring prior to Aβ deposition and neuroinflammatory responses [9], 10]. Mitochondria play a central role in maintaining neuronal energy homeostasis, regulating ROS production and calcium signaling, as well as controlling apoptotic pathways [11]. Studies have shown that Aβ_1–42_ can disrupt mitochondrial membrane potential (ΔΨm), impair oxidative phosphorylation, and inhibit mitophagy, leading to the accumulation of damaged mitochondria, decreased ATP production, and increased ROS levels [12], 13]. These changes form a vicious cycle of metabolic stress that accelerates neuronal degeneration.

In recent years, natural compounds have gained increasing attention in AD research due to their chemical diversity and multi-target mechanisms [14]. Aloe emodin, an anthraquinone compound derived from traditional medicinal plants such as *Rheum palmatum* and *Aloe vera*, exhibits a variety of biological activities, including anti-inflammatory, antioxidant, antifibrotic, and autophagy-regulating effects [15], [16], [17]. Previous studies have shown that aloe emodin can activate the AMPK/PGC-1α/SIRT3 signaling pathway to promote mitophagy, thereby alleviating cognitive deficits and neuroinflammation in AD mouse models [18]. In addition, aloe emodin has been reported to suppress NLRP3 inflammasome activation and pyroptosis, providing tissue-protective effects in models of cerebral ischemia, and radiation-induced cardiomyopathy [15], 19]. However, whether aloe emodin can protect neurons from Aβ-induced toxicity remains largely unexplored.

Although aloe emodin has been reported to exert neuroprotective effects in AD models, the specific molecular target and underlying mechanism by which aloe emodin antagonizes Aβ_1–42_-induced neurotoxicity remain largely unknown. Based on bioinformatics prediction and preliminary analysis, we hypothesized that aloe emodin attenuates Aβ_1–42_-induced neuronal injury by targeting and downregulating protein phosphatase Mg^2+^/Mn^2+^ dependent 1 K (PPM1K) expression. The present study aimed to answer the following research question: can aloe emodin protect SH-SY5Y cells against Aβ_1–42_-induced neurotoxicity by inhibiting PPM1K?

## Materials and methods

### Cell culture and treatment

The human neuroblastoma cell line SH-SY5Y was obtained from Wuhan Yousi Biotechnology Co., Ltd. (YLH254, Wuhan, China), and cultured in SH-SY5Y-specific medium at 37 °C in a humidified incubator with 5 % CO_2_.

Aloe emodin and Aβ_1–42_ were purchased from Sigma-Aldrich (A7687-25 MG, ≥95 % HPLC, USA) and prepared as stock solutions according to the manufacturer’s instructions. For model induction, cells were treated with 10 μM Aβ_1–42_ for 24 h. For aloe emodin intervention, cells were co-treated with 10 μM Aβ_1–42_ and aloe emodin at concentrations of 2, 4, or 6 μM for 24 h. Subsequent assays were then performed as described.

### Cell transfection

The PPM1K overexpression plasmid (OE-PPM1K) and the empty vector control (OE-NC) were obtained from GeneUniversal Biotechnology (Anhui, China). For transfection, SH-SY5Y cells were seeded into 6-well plates and transfected using Lipofectamine 3000 (Invitrogen, USA) according to the manufacturer’s instructions. After 48 h, the medium was replaced, and cells were subjected to subsequent treatments or harvested for further analysis.

### Bioinformatics analysis

To identify AD-related genes, genes associated with Alzheimer’s disease were retrieved from GeneCards (https://www.genecards.org) and Comparative Toxicogenomics Database (CTD, https://ctdbase.org) using the keyword “Alzheimer’s disease”. For GeneCards, genes were sorted by the database-calculated relevance score in descending order. For CTD, genes were sorted by the database-annotated inference score in descending order. Gene expression profiles were obtained from two independent GEO datasets: GSE207821 and GSE267554 (human AD brain tissues). Differential expression analysis was performed using the GEO2R online tool with Benjamini–Hochberg multiple testing correction. Cutoff criteria were set as |log_2_FC|>1 and adjusted p-value (FDR) <0.05. Additionally, the BATMAN-TCM database (http://bionet.ncpsb.org.cn/batman-tcm) was used to predict potential targets of aloe emodin. Targets with a prediction score ≥20 were selected for subsequent intersection and analysis.

### Molecular docking analysis

To evaluate the binding affinity between aloe emodin and the potential target, the 3D structure of aloe emodin (CID: 10207) was retrieved from the PubChem database (https://pubchem.ncbi.nlm.nih.gov), and energy minimization along with format conversion was performed using AutoDockTools 1.5.6. The crystal structure of the PPM1K protein was obtained from the Protein Data Bank (PDB) database. Molecular docking was then carried out using AutoDock Vina to assess the interaction and binding affinity between aloe emodin and PPM1K. The docking results were visualized in three dimensions using PyMOL 2.5.0 software.

### Cell viability assay

Cell viability was assessed using a Cell Counting Kit-8 (CCK-8, C0038, Beyotime, Shanghai, China). SH-SY5Y cells were seeded into 96-well plates at a density of 1 × 10^4^ cells per well and treated according to experimental groups for 24 h. Subsequently, 10 μL of CCK-8 working solution was added to each well, followed by incubation at 37 °C for 2 h. The absorbance was measured at 450 nm using a microplate reader.

### Flow cytometry for apoptosis detection

Cell apoptosis was assessed using an Annexin V-FITC/PI Apoptosis Detection Kit (556,547, BD, USA). After treatment, cells were harvested and resuspended in binding buffer. Then, 5 μL of Annexin V-FITC and 5 μL of propidium iodide (PI) were added to each sample, followed by incubation in the dark for 15 min at room temperature. Apoptotic cells were analyzed using a CytoFLEX flow cytometer (Beckman Coulter), and the data were processed using FlowJo software.

### ROS detection

Intracellular ROS levels were measured using the DHE fluorescent probe (S0033, Beyotime, China). After treatment, cells were washed with PBS and incubated with 10 μM DHE working solution at 37 °C for 30 min in the dark. Fluorescence images were captured using a fluorescence microscope, and fluorescence intensity was quantified using ImageJ software.

### ELISA assay

Cell culture supernatants were collected and analyzed using commercial ELISA kits to quantify the levels of TNF-α, IL-1β, IL-6, MDA, and GSH, as well as the enzymatic activity of SOD, in accordance with the manufacturer’s instructions.

### Real-time quantitative PCR (RT-qPCR)

Total RNA was extracted from cells using TRIzol reagent (ELK Biotechnology, China), and reverse-transcribed into cDNA using a commercial cDNA synthesis kit (ELK Biotechnology, China). Quantitative PCR was performed using SYBR Green PCR SuperMix (ELK Biotechnology, China), with β-actin used as the internal control. Relative gene expression was calculated using the 2^−ΔΔCt^ method. Primer sequences are listed in Table 1.

**Table 1:** The RT-qPCR primer sequences.

Gene		Primer (5–3′)
H-β-actin	Sense	GTC​CAC​CGC​AAA​TGC​TTC​TA
	Antisense	TGC​TGT​CAC​CTT​CAC​CGT​TC
H-PPM1K	Sense	TGA​TGC​AAC​TCT​TCT​GAC​CTC​TG
	Antisense	TAT​GGT​CAA​TGG​TCA​GCT​TCA​TG

### Western blot assay

Total protein was extracted from cells and separated by SDS-PAGE, followed by transfer onto PVDF membranes. After blocking with 5 % non-fat milk for 1 h at room temperature, the membranes were incubated overnight at 4 °C with primary antibodies against PPM1K (ab224424, dilution rate 1:1,000, Abcam), Caspase-3 (ab32351, dilution rate 1:1,000, Abcam), Cleaved caspase-3 (AF7022, dilution rate 1:500, Affbiotech), and β-actin (TDY051, dilution rate 1:10,000, Beijing TDY Biotech Co.,Ltd). The next day, membranes were incubated with HRP-conjugated secondary antibody (AS1107, dilution rate 1:10,000, ASPEN) at room temperature for 1 h. Protein bands were visualized using enhanced chemiluminescence (ECL), and band intensities were quantified using ImageJ software.

### Statistical analysis

All experiments were performed with three independent biological replicates (cells from different passages), and each biological replicate included three technical replicates within the same culture plate. Statistical analyzes were performed using GraphPad Prism 8. Before statistical analysis, data normality was assessed. Differences between multiple groups were evaluated by one-way analysis of variance (ANOVA) followed by Tukey’s post-hoc test, and differences between two groups were analyzed using unpaired student’s *t*-test. The correction for multiple comparisons was applied to control false positive errors. Data are presented as mean ± standard deviation (SD). A p-value of <0.05 was considered statistically significant.

### Ethics statement

This study only employed *in vitro* cell line experiments (SH-SY5Y cells). No human participants, human samples, clinical data, or experimental animals were involved in this research. Therefore, ethical approval and informed consent are not applicable for this study.

## Results

### Aloe emodin exerts no toxic effects on SH-SY5Y cells

**Figure 1:**
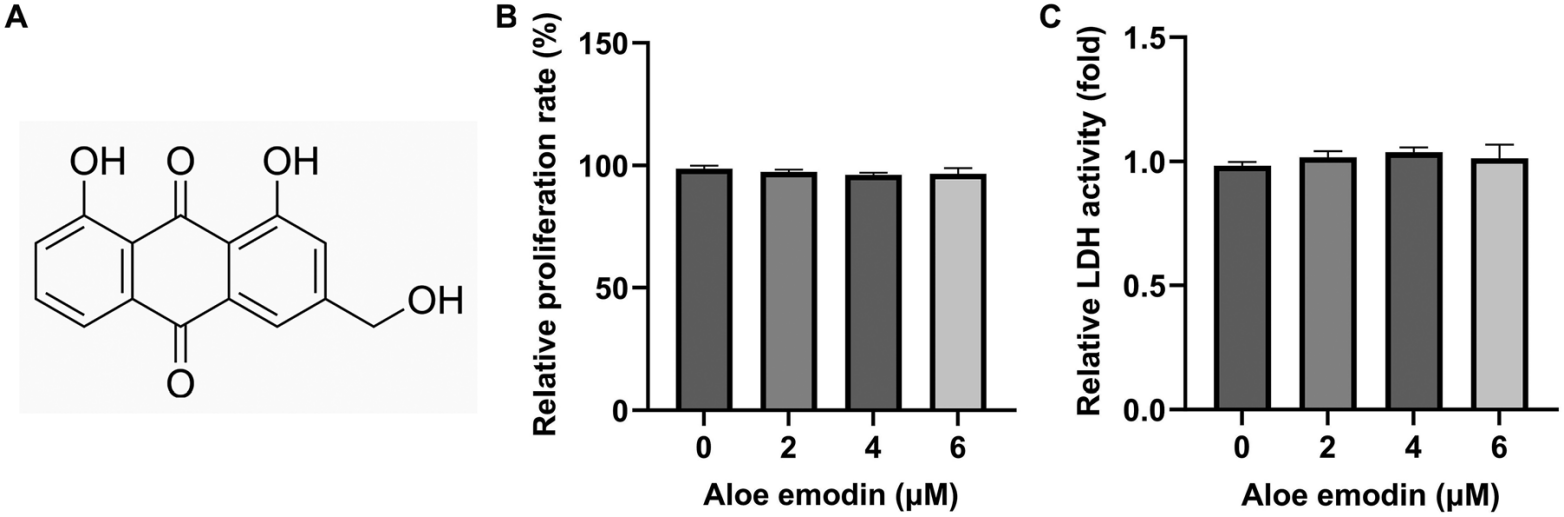
Aloe emodin exerts no toxic effects on SH-SY5Y cells. (A) The chemical molecular formula of aloe emodin. The effects of aloe emodin on SH-SY5Y cell proliferation activity (B) and LDH activity (C).

Figure 1A shows the chemical molecular formula of aloe emodin. The results showed that aloe emodin had no significant effect on cell proliferation activity (Figure 1B) or LDH activity (Figure 1C), suggesting that aloe emodin has no cytotoxic effects.

### Aloe emodin alleviates Aβ_1–42_-induced injury in SH-SY5Y cells

**Figure 2:**
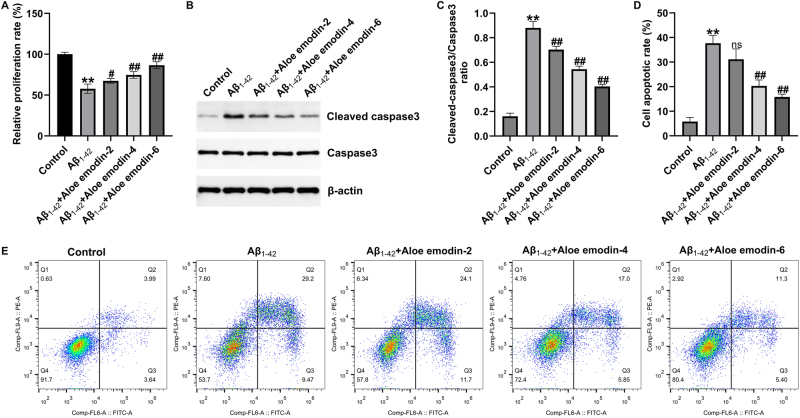
Aloe emodin alleviates Aβ_1–42_-induced apoptosis in SH-SY5Y cells. (A) Cell viability assessed by the CCK-8 assay; (B) western blot analysis of Caspase3 and cleaved caspase3 expression; (C) quantitative analysis of the cleaved caspase3/Caspase3 ratio. (D and E) Cell apoptosis assessed by flow cytometry. Data are presented as mean ± SD (n=3). **p<0.01 vs. Control group; #, ##p<0.05, 0.01, vs. Aβ_1–42_ group.

**Figure 3:**
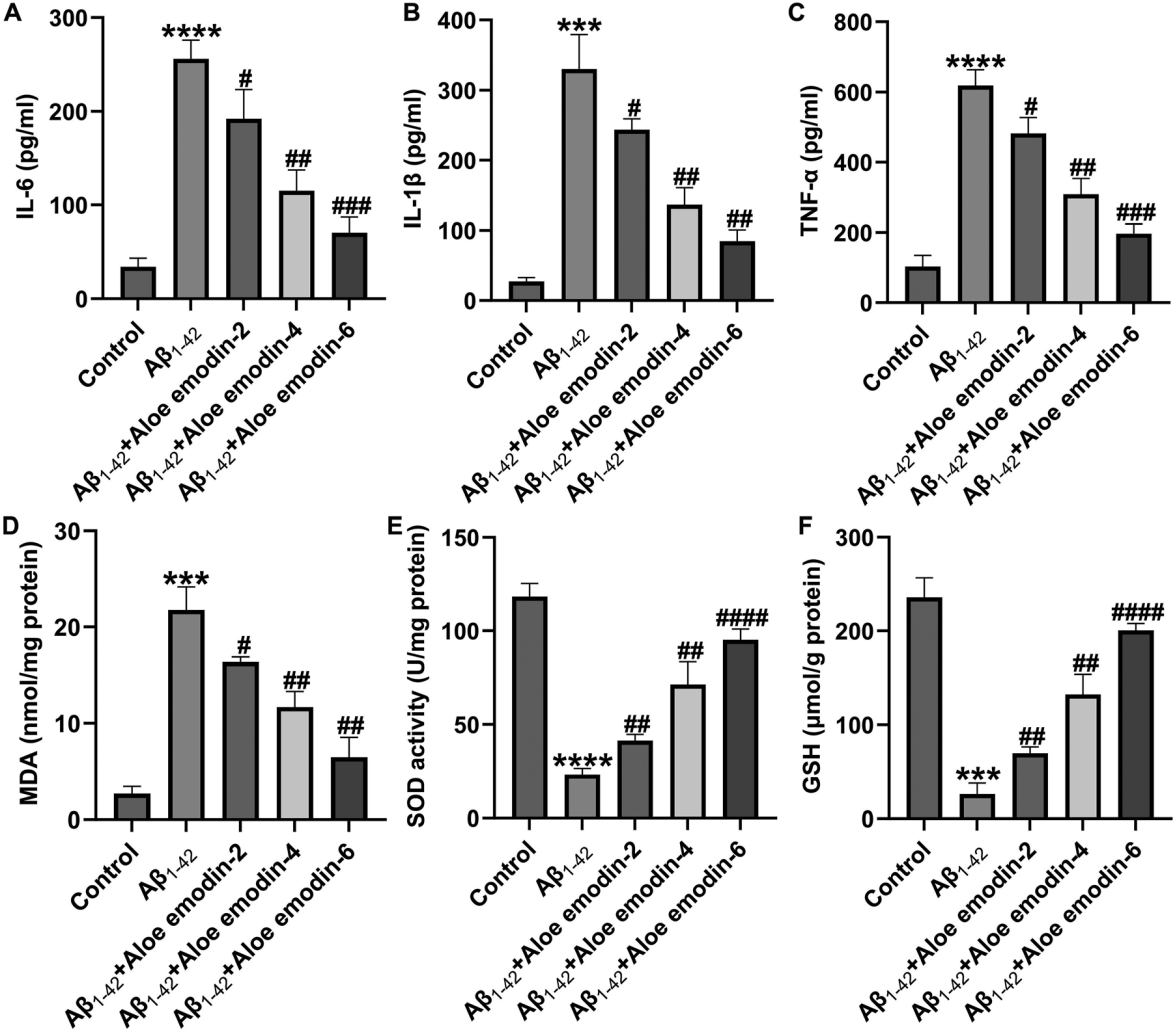
Aloe emodin alleviates Aβ_1–42_-induced inflammatory response and oxidative stress in SH-SY5Y cells. (A–C) ELISA detection of inflammatory cytokines IL-6, IL-1β, and TNF-α in the culture supernatant; (D–F) quantification of MDA content, SOD activity, and GSH levels; data are presented as mean ± SD (n=3). ***, ****p<0.001, 0.0001 vs. Control group; #, ##, ###, ####p<0.05, 0.01, 0.001, 0.0001 vs. Aβ_1–42_ group.

**Figure 4:**
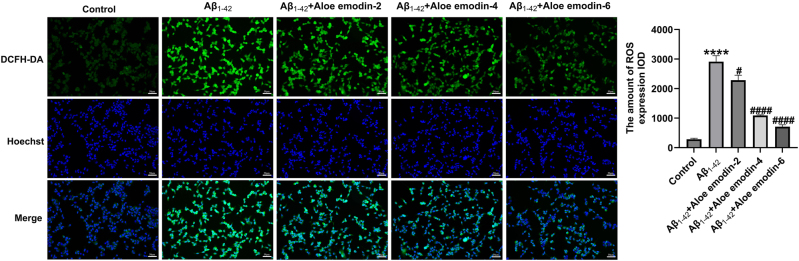
Aloe emodin reduces Aβ_1–42_-induced ROS production in SH-SY5Y cells. Intracellular ROS levels were detected using a fluorescence probe and quantified. Data are presented as mean ± SD (n=3). *p<0.05 vs. Control group; #p<0.05, vs. Aβ_1–42_ group. ****p<0.0001 vs. Control group; #, ####p<0.05, 0.0001 vs. Aβ_1–42_ group.

To evaluate the neuroprotective effect of aloe emodin, an Aβ_1–42_-induced injury model was established using human neuroblastoma SH-SY5Y cells. Exposure to 10 μM Aβ_1–42_ for 24 h significantly reduced cell viability (Figure 2A). Based on this model, cells were treated with aloe emodin at concentrations of 2, 4, and 6 μM for 24 h to assess the protective effects. CCK-8 assay results showed that aloe emodin significantly improved cell viability in a dose-dependent manner, with the 4 and 6 μM treatment groups exhibiting a notable increase compared to the model group (Figure 2A). Western blot analysis revealed that Aβ_1–42_ increased the expression of cleaved caspase3 (a key apoptotic marker), while aloe emodin treatment notably reduced the cleaved caspase3/caspase3 ratio (Figure 2B and C), indicating that aloe emodin inhibits cell apoptosis. Flow cytometry results further showed that Aβ_1–42_ markedly induced apoptosis in SH-SY5Y cells, whereas aloe emodin treatment effectively reduced the proportion of apoptotic cells, with the strongest effect observed at 6 μM (Figure 2D and E). ELISA results demonstrated that Aβ_1–42_ significantly increased the secretion of inflammatory cytokines IL-6, IL-1β, and TNF-α, while aloe emodin intervention markedly suppressed their levels (Figure 3A–C). Moreover, aloe emodin treatment reduced the accumulation of malondialdehyde (MDA) and significantly enhanced the activity of superoxide dismutase (SOD) and the level of glutathione (GSH) (Figure 3D–F). ROS fluorescence assay results showed that aloe emodin significantly inhibited intracellular ROS generation induced by Aβ_1–42_ (Figure 4). These findings suggest that aloe emodin protects SH-SY5Y cells from Aβ_1–42_-induced injury by attenuating apoptosis, inflammation, and oxidative stress.

### PPM1K as a potential target of aloe emodin in AD

**Figure 5:**
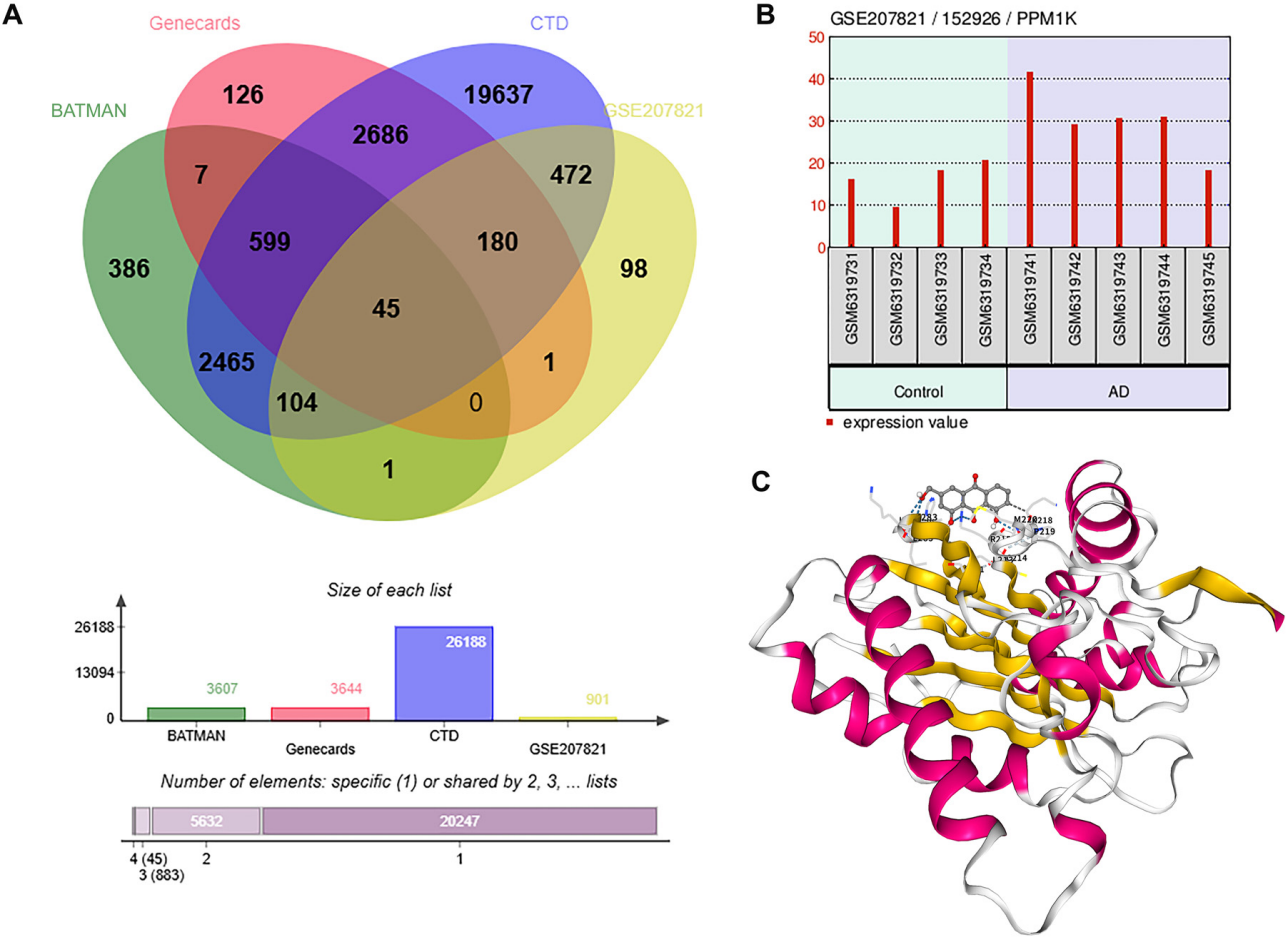
PPM1K as a potential target of aloe emodin. (A) Venn diagram showing the intersection of AD-related genes (GeneCards + CTD), differentially expressed genes in two independent GEO datasets (GSE207821), and predicted targets of aloe emodin from BATMAN-TCM; a total of 45 candidate genes were identified; (B) PPM1K is significantly upregulated in brain tissues of AD patients in GSE207821 (adjusted p=1.13e−03) and GSE267554 (adjusted p=2.66e^−05^); (C) molecular docking predicted a potential binding mode between aloe emodin and PPM1K. Binding affinity: −7.0 kcal/mol.

To identify potential targets of aloe emodin in AD, we integrated data from GeneCards, CTD, and the GSE207821 and GSE267554 expression dataset to screen for differentially expressed AD-related genes. Meanwhile, BATMAN-TCM was used to predict the potential protein targets of aloe emodin. By intersecting gene sets from all four sources, 45 candidate genes were obtained (Figure 5A). PPM1K was selected from the 45 candidates because it was significantly upregulated in AD brain tissues, is functionally associated with mitochondrial dysfunction and neuronal injury, exhibits predicted binding potential with aloe emodin, and has been reported to participate in neurological damage. Among the 45 candidate genes, PPM1K was significantly upregulated in brain tissues of AD patients in both GSE207821 (adjusted p=1.13e−03, Figure 5B, Supplementary GSE207821.top) and the independent validation dataset GSE267554 (adjusted p=2.66e^−05^, Supplementary Figure S1, Supplementary GSE267554.top). Although the sample size of this dataset is relatively small, published studies have confirmed that PPM1K is significantly upregulated in multiple neuronal injury models, and is closely associated with mitochondrial dysfunction, oxidative stress and neuronal death [20], 21]. Therefore, PPM1K was selected as the key target for further experimental verification. Further molecular docking analysis predicted a potential in silico interaction between aloe emodin and PPM1K, with a binding affinity of −7.0 kcal/mol (Figure 5C). These in silico results suggest that PPM1K is a putative molecular target worthy of further experimental validation for mediating the neuroprotective effects of aloe emodin.

### Aloe emodin modulates PPM1K in Aβ_1–42_-induced SH-SY5Y cells

**Figure 6:**
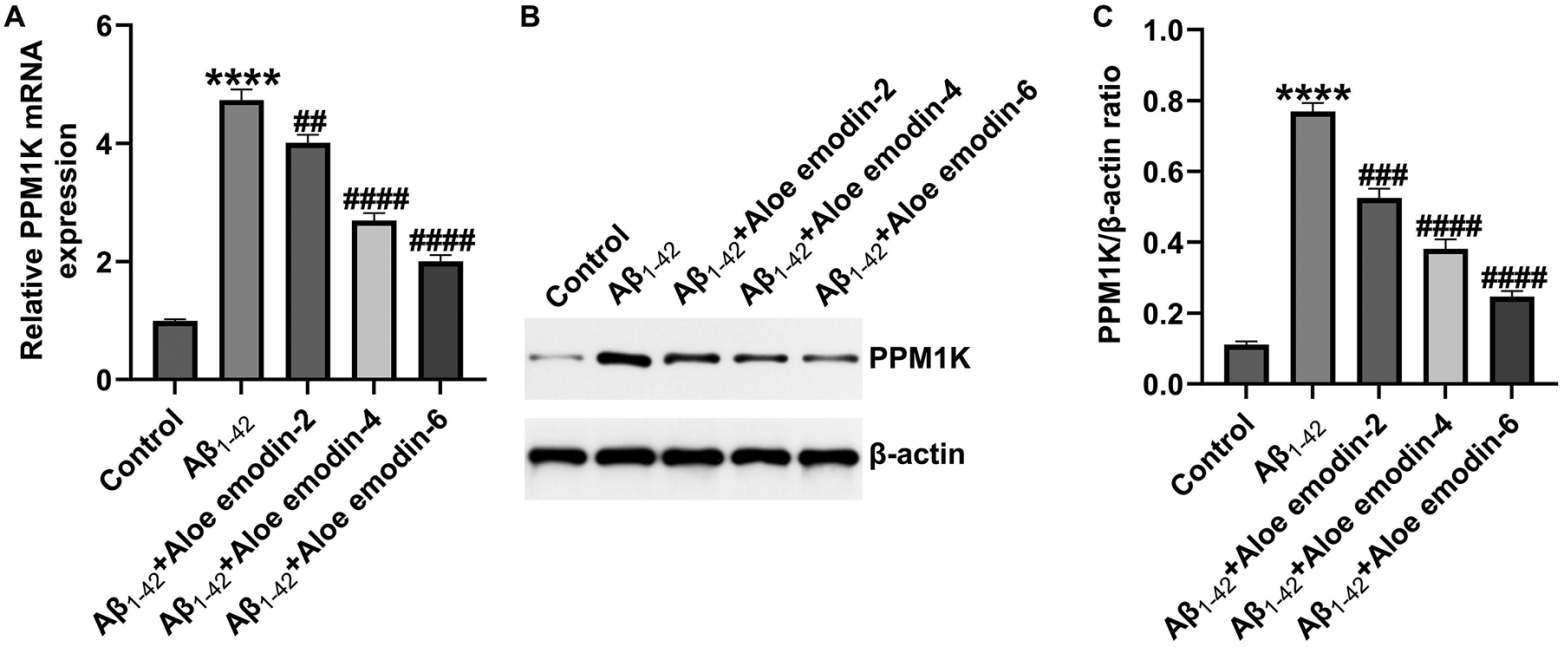
Aloe emodin modulates PPM1K expression in Aβ_1–42_-induced SH-SY5Y cells. (A) RT-qPCR analysis of PPM1K mRNA expression in different treatment groups; (B–C) western blot analysis and quantification of PPM1K protein levels. Data are presented as mean ± SD (n=3). ****p<0.0001 vs. Control group; ##, ###, ####p<0.01, 0.001, 0.0001 vs. Aβ_1–42_ group. Relative expression of mRNA and protein was normalized to the internal control β-actin and calculated using the 2^−ΔΔCt^ method (RT-qPCR) or densitometric quantification relative to β-actin (western blot assay).

As shown in Figure 6A, Aβ_1–42_ stimulation significantly upregulated PPM1K mRNA expression, whereas aloe emodin treatment dose-dependently suppressed this expression. Consistently, western blot assay revealed that aloe emodin effectively attenuated the Aβ_1–42_-induced increase in PPM1K protein levels (Figures 6B and C).

### Aloe emodin attenuates Aβ_1–42_-induced injury in SH-SY5Y cells via regulation of PPM1K

**Figure 7:**
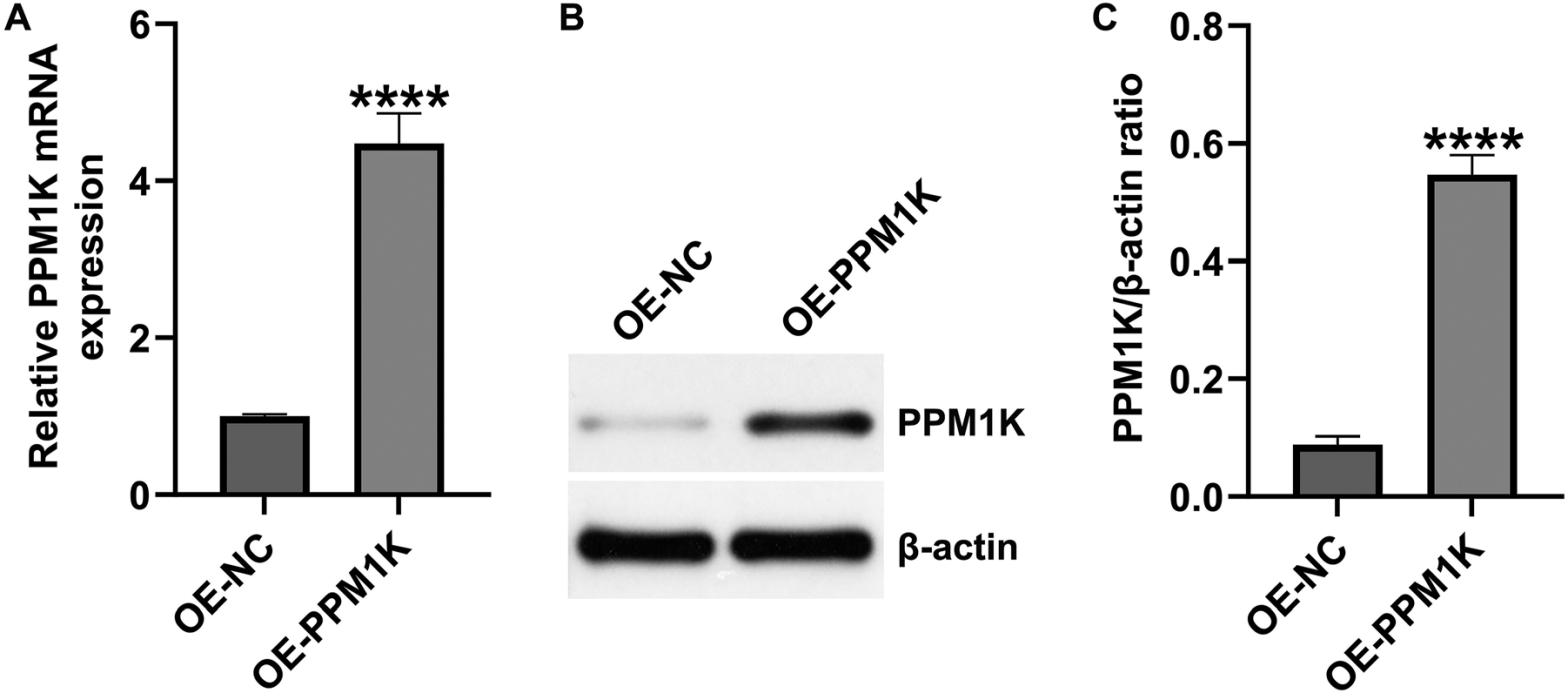
Transfection efficiency of OE- PPM1K in SH-SY5Y cells. SH-SY5Y cells were transfected with OE-NC or OE- PPM1K for 24 h. (A) RT-qPCR analysis of PPM1K mRNA expression in different treatment groups; (B–C) western blot analysis and quantification of PPM1K protein levels. Data are presented as mean ± SD (n=3). ****p<0.0001 vs. OE-NC group. Relative expression of mRNA and protein was normalized to the internal control β-actin and calculated using the 2^−ΔΔCt^ method (RT-qPCR) or densitometric quantification relative to β-actin (western blot assay).

**Figure 8:**
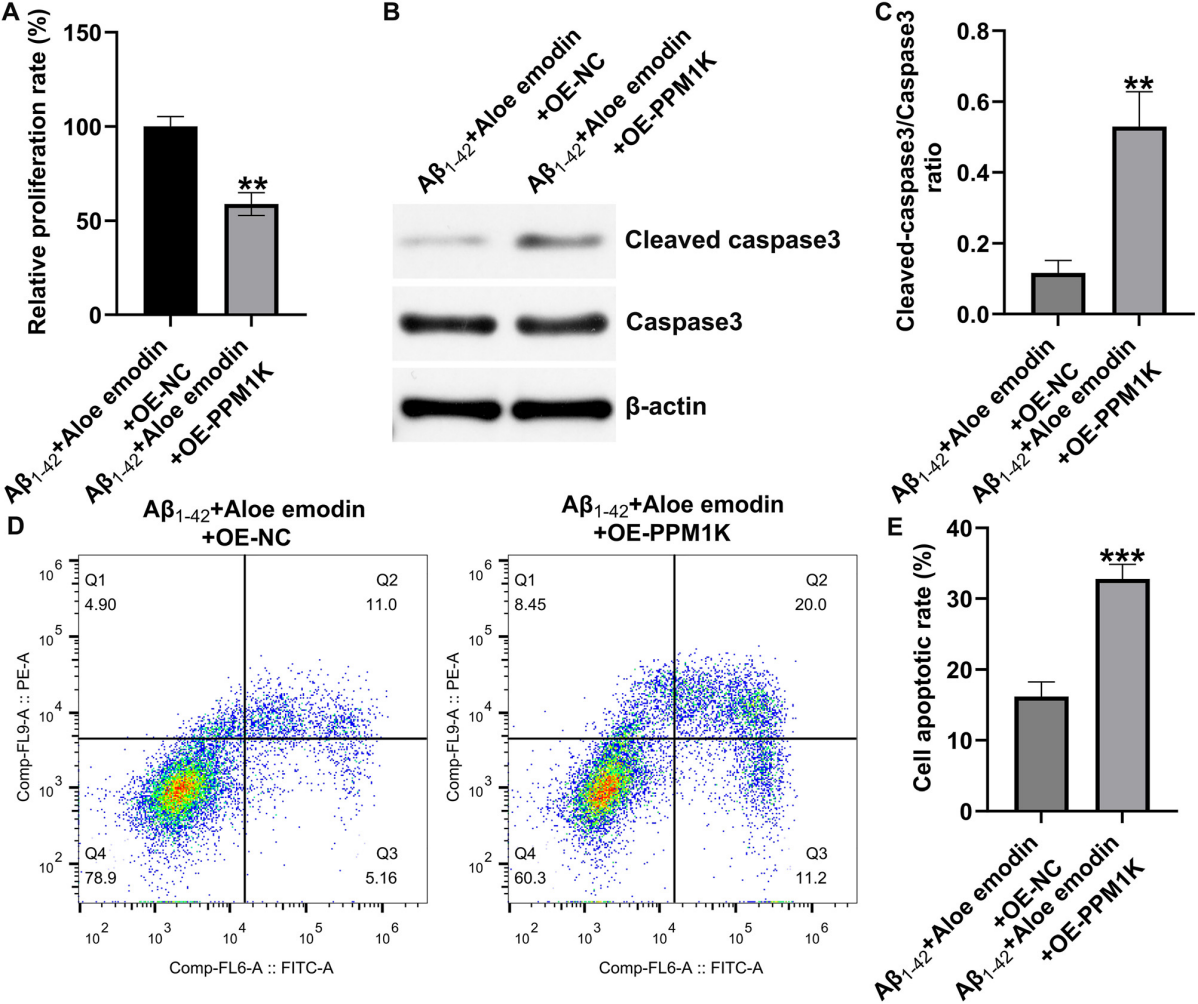
Aloe emodin alleviates Aβ_1–42_-induced apoptosis in SH-SY5Y cells via regulation of PPM1K. (A) Cell viability measured by CCK-8 assay; (B and C) western blot analysis of cleaved caspase3 and Caspase3, and quantification of their ratio. (D and E) Cell apoptosis assessed by flow cytometry. Data are presented as mean ± SD (n=3). **, ***p<0.01, 0.001 vs. Aβ_1–42_ + aloe emodin + OE-NC group.

**Figure 9:**
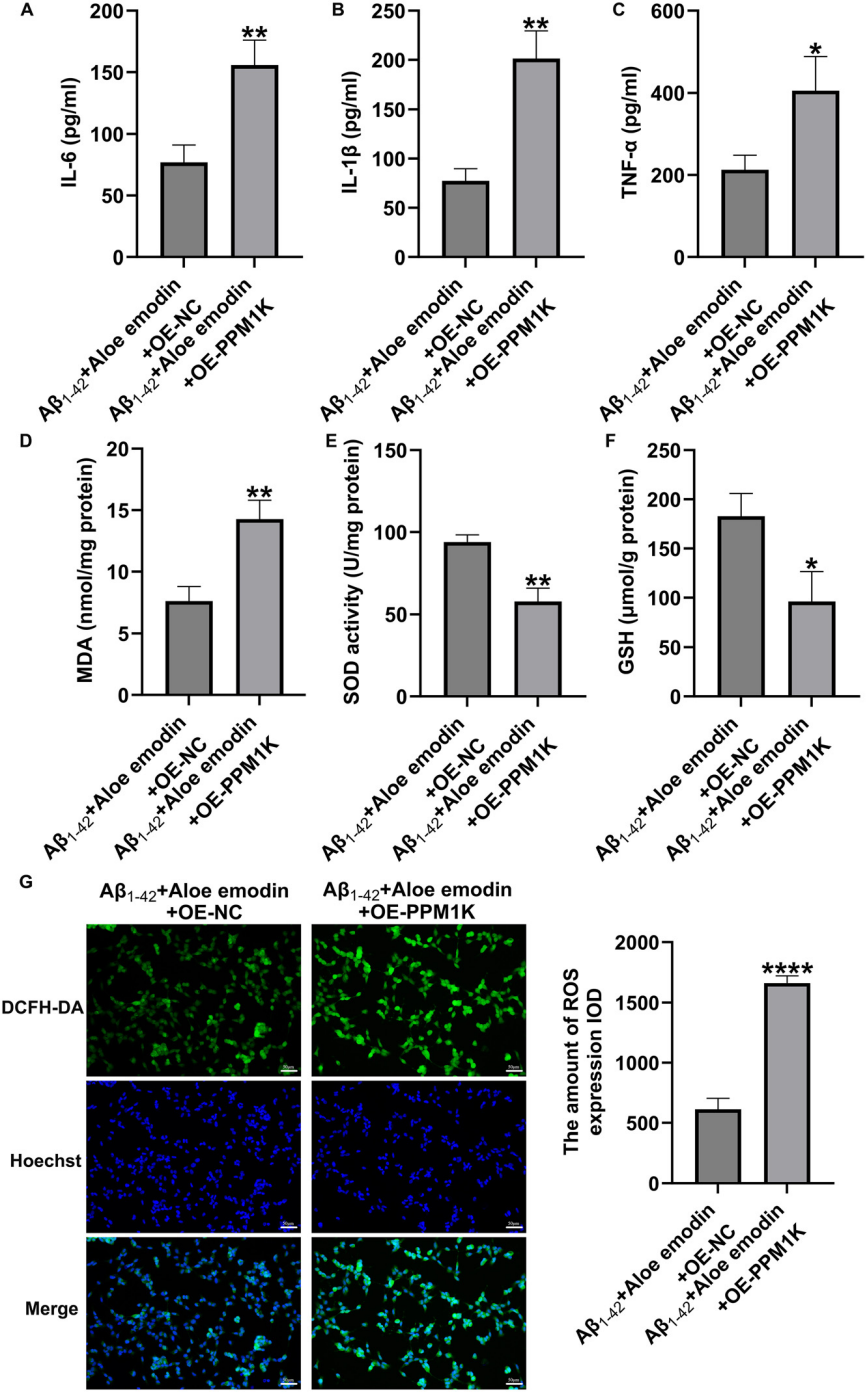
Aloe emodin alleviates Aβ_1–42_-induced inflammatory response and oxidative stress in SH-SY5Y cells via regulation of PPM1K. (A–C) ELISA detection of IL-6, IL-1β, and TNF-α levels in the culture supernatant; (D–F) measurement of MDA content, SOD activity, and GSH levels. (G) Intracellular ROS production assessed by fluorescence probe and quantified. Data are presented as mean ± SD (n=3). *, **, ****p<0.05, 0.01, 0.0001 vs. Aβ_1–42_ + aloe emodin + OE-NC group.

To investigate the role of PPM1K in the protective effects of aloe emodin against Aβ_1–42_-induced injury in SH-SY5Y cells, we overexpressed PPM1K by plasmid transfection (Figure 7A–C). Aloe emodin significantly alleviated the Aβ_1–42_-induced reduction in cell viability. However, this protective effect was markedly diminished by PPM1K overexpression (Figure 8A). Western blot analysis further revealed that PPM1K overexpression restored caspase3 activation, as indicated by an increased ratio of cleaved caspase3/caspase3 ratio, thereby partially reversing the anti-apoptotic effect of aloe emodin (Figure 8B and C). Flow cytometry analysis further showed that PPM1K overexpression significantly increased the proportion of both early and late apoptotic cells under Aβ_1–42_ and aloe emodin co-treatment conditions (Figure 8D and E). Moreover, ELISA results demonstrated that PPM1K overexpression reversed the anti-inflammatory effects of aloe emodin, as evidenced by elevated levels of IL-6, IL-1β, and TNF-α (Figure 9A–C). In terms of oxidative stress, PPM1K upregulation markedly increased MDA levels and reduced SOD activity and GSH content (Figure 9D–F). This was accompanied by enhanced intracellular ROS accumulation in aloe emodin-treated cells exposed to Aβ_1–42_ (Figure 9G). These results indicate that PPM1K plays a critical regulatory role in modulating the neuroprotective effects of aloe emodin against Aβ_1–42_-induced cellular injury.

## Discussion

In the present study, we found that aloe emodin attenuates Aβ_1–42_-induced neurotoxicity in SH-SY5Y cells via downregulation of PPM1K. In silico molecular docking suggested a potential interaction between aloe emodin and PPM1K, which was further supported by cellular functional assays.

AD is a neurodegenerative disorder characterized by abnormal Aβ deposition, neuronal loss, chronic neuroinflammation, and impaired energy metabolism [13]. Given the complex and multifactorial nature of AD pathogenesis, single-target interventions often yield limited therapeutic benefits. Therefore, identifying potential therapeutic targets capable of simultaneously regulating metabolic homeostasis, inflammatory responses, and cell fate is of great significance.

By integrating data from GeneCards, CTD, GEO (GSE207821), and BATMAN-TCM databases, we identified that PPM1K is significantly upregulated in the brain tissues of AD patients. PPM1K is a serine/threonine protein phosphatase localized in the mitochondrial matrix, primarily responsible for regulating the catabolism of branched-chain amino acids (BCAAs) through the dephosphorylation of the branched-chain α-keto acid dehydrogenase (BCKD) complex, thereby maintaining mitochondrial energy homeostasis [21], 22]. Mu et al. found that impaired BCAA catabolism regulated by PPM1K contributes to the pathogenesis of polycystic ovary syndrome (PCOS) [23]. Studies have shown that PPM1K influences the development of leukemia by regulating CDC20-mediated ubiquitination of MEIS1 [24]. In addition, PPM1K has been shown to regulate cerebral ischemia-reperfusion injury by promoting ferroptosis in neurons [20]. As mitochondrial dysfunction is considered an early pathological event in AD [10], the aberrant upregulation of PPM1K may exacerbate metabolic stress and enhance neuronal susceptibility to Aβ-induced toxicity.

In the Aβ_1–42_-induced SH-SY5Y cell model, we confirmed that Aβ_1–42_ significantly reduced cell viability, promoted apoptosis, induced the release of pro-inflammatory cytokines, and triggered ROS production-consistent with previous findings on Aβ-induced neurotoxicity [25]. Treatment with aloe emodin markedly ameliorated these pathological changes. However, overexpression of PPM1K significantly attenuated the protective effects of aloe emodin, suggesting that PPM1K plays a critical regulatory role in aloe emodin-mediated neuroprotection. These cellular functional results fuether support the bioinformatic predictions and suggest that PPM1K may serve as a potential therapeutic target for AD intervention, whereas *in vitro* binding assays are still needed to confirm direct interaction.

PPM1K-mediated disruption of BCAA metabolism can directly impair the efficiency of mitochondrial oxidative phosphorylation, leading to energy crisis and excessive ROS accumulation [26], 27]. Moreover, mitochondrial metabolic stress has been identified as a key trigger for NLRP3 inflammasome activation, and NLRP3-mediated pyroptosis plays a critical role in AD progression [28], 29]. Although NLRP3-associated molecules were not directly examined in this study, aloe emodin significantly suppressed the release of pro-inflammatory cytokines and reduced oxidative stress levels-suggesting that the anti-inflammatory effects may be partially mediated through PPM1K-dependent regulation of metabolic stress and indirect inhibition of inflammasome activation.

Moreover, PPM1K may also participate in the regulation of neuronal injury by modulating the AMPK signaling network. As a cellular energy sensor, AMPK activation has been shown to suppress oxidative stress, promote mitophagy, and alleviate Aβ-induced toxicity [30], 31]. Previous studies have demonstrated that aloe emodin activates the AMPK/PGC-1α/SIRT3 pathway to improve mitochondrial function and enhance mitophagy [18]. In combination with our findings, we speculate that PPM1K may act as an upstream regulatory node in this signaling axis, and its inhibition may help restore AMPK-mediated protective metabolic responses.

Although this study provides preliminary evidence that aloe emodin mitigates Aβ-induced neurotoxicity by targeting PPM1K, several limitations should be noted. First, this study only conducted PPM1K overexpression experiments without knockdown or knockout validation, and lacked *in vivo* verification using PPM1K gene-edited animal models, which limits the systematic confirmation of its role in AD pathology. Second, *in vitro* biochemical validation (e.g., pull-down, CETSA, MST, SPR) was not performed to confirm the direct physical interaction between aloe emodin and PPM1K, and molecular docking only provides in silico predictive evidence; meanwhile, the absence of transcriptome enrichment analysis and direct detection of mitochondrial metabolic indicators restricts the clarification of PPM1K downstream pathways and its specific mechanism in regulating Aβ neurotoxicity. Third, plasmid-mediated overexpression may cause off-target effects, which may interfere with the accurate interpretation of aloe emodin’s neuroprotective action. Fourth, only the canonical PPM1K full-length isoform was used; the functions of other transcript variants remain unclear. Fifth, the research was limited to SH-SY5Y cells, without primary neurons or glial cell models.

Future work will integrate multi-omics analysis to assess off-target effects and clarify the functions of different PPM1K isoforms. Mitochondrial function and metabolic tests will be supplemented to improve mechanistic depth. Further validation will be performed in primary neurons, independent human datasets and AD animal models. Advanced spatial multi-omics approaches, including spatial transcriptomics and high-plex spatial CITE-seq, will be applied to explore neuroinflammation and the cellular characteristics of PPM1K [32], 33].

In conclusion, this study demonstrates that aloe emodin alleviates Aβ_1–42_-induced neuronal injury at least partly by down-regulating PPM1K expression, thereby reducing ROS production, inhibiting apoptosis, and alleviating inflammation. These findings offer new insights into the mechanism by which natural compounds may intervene in AD and lay the groundwork for further exploration of PPM1K as a potential therapeutic target for AD.

## Supplementary Material

Supplementary Material

Supplementary Material

Supplementary Material

Supplementary Material
