# Supplementary material for: Aloe emodin attenuates Aβ1–42-Induced neurotoxicity in SH-SY5Y cells via downregulation of PPM1K
Source: Open Med (Wars). 2026 Jul 7;21(1):20261473. doi: 10.1515/med-2026-1473 (PMC13340830; doi:10.1515/med-2026-1473)
Supplement: Supplementary file 4 — Supplementary Material [file j_med-2026-1473_suppl_004.docx]

Figure S1. PPM1K is significantly upregulated in brain tissues of AD patients in GSE267554 (adjusted P = 2.66e-05).
